# Supplementary material for: Colloidal Stability of Silica-Modified Magnetite Nanoparticles: Comparison of Various Dispersion Techniques
Source: Nanomaterials (Basel). 2021 Dec 4;11(12):3295. doi: 10.3390/nano11123295 (PMC8708453; doi:10.3390/nano11123295)
Supplement: Supplementary file 1 [file nanomaterials-11-03295-s001.zip › nanomaterials-1447452-supplementary.pdf]

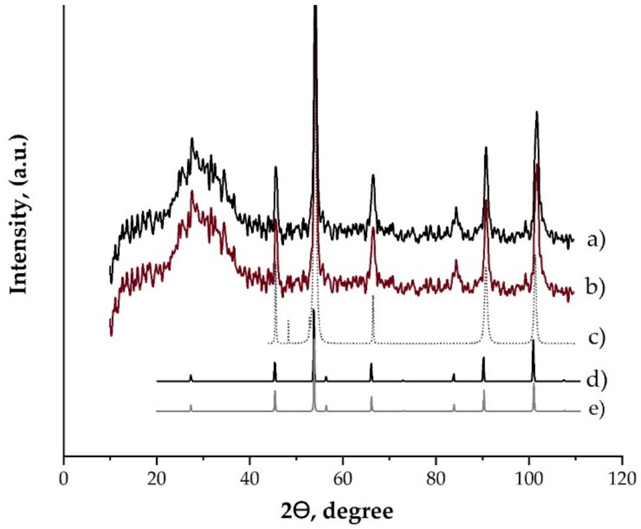

Figure S1. X-ray powder diffraction (XRD) of a) Fe_3O_4, b) Fe_3O_4/APTES, c) calculated data for Fe_3O_4, d) Fe_3O_4, and e) Fe_2O_3 patterns from the Crystallography Open Database

The lattice parameters determined for all samples fall within the values range typical of maghemite and magnetite, respectively 8.33–8.34 Å (ICDD - PDF 39–1346) and 8.396–8.400 Å (ICDD - PDF 19–629. This phenomenon can be related to the oxidation of Fe^{2+} either during sample drying or modification, which leads to the formation of non-stoichiometric $Fe_{3-\delta}O_4$ with δ varying from zero (*stoichiometric magnetite*) to 1/3 (*completely oxidized*). [42]. Stoichiometric magnetite contains an ideal amount of Fe^{2+} based on the formula Fe_3O_4 and it has a value of x = 0.50. During the oxidation of magnetite, the Fe^{2+}/Fe^{3+} ratio (Equation 1) decreases and x becomes less than 0.50. This form is considered as a nonstoichiometric or partially oxidized magnetite [42]. Stoichiometry can be easily expressed as ratio as follows:

$$x = \frac{Fe^{2+}}{Fe^{3+}} = \frac{1-3\delta}{2+2\delta}, \quad (1)$$

In conclusion, the composition of the crystalline component of the samples can be allocated as follows: $Fe_{2,94}O_4$ and $Fe_{2,88}O_4$ for the Fe_3O_4 and Fe_3O_4/APTES samples, respectively (Table 1). The decrease in the content of stoichiometric magnetite after APTES modification can be explained by the oxidation of magnetite during deposition.

Table S1. Microstructure of MNPs

Sample	Fe_3O_4			Fe_3O_4/APTES		
hkl	2θ, °	d, Å	FWHM	2θ, °	d, Å	FWHM
220	45.45	2.965	0.636(8)	45.45	2.959	0.504(9)
311	53.90	2.527	0.662(2)	53.95	2.525	0.679(5)
400	66.30	2.095	0.780(1)	66.3	2.095	0.890(4)
422	83.85	1.714	0.975(2)	84.05	1.711	0.844(9)
511	90.70	1.610	0.940(7)	90.75	1.609	0.828(5)
440	101.35	1.481	0.899(1)	101.55	1.479	1.026(6)
a, A	8.3813			8.3789		
X	0.37			0.290		
δ	0.069			0.117		
Structure	$Fe_{2,93}O_4$			$Fe_{2,88}O_4$		
% Fe_3O_4	78.8			63.7		
D_{XRD}, nm	17.1±2.3			20.5±3.3		

CV, %	13.5	16.1

d - interplanar distance, Å
Q - angle at which the reflex was measured, °
FWHM - full width at half maximum of XRD reflex, °
a - interplanar distance, Å
X - the Fe^{2+}/Fe^{3+} ratio
δ – calculated value, which range from zero (stoichiometric magnetite) to 1/3 (completely oxidized)
D_{XRD} - average particle size calculated by the Scherrer equation ± standard deviation, nm
CV - coefficient of variation characterizing the polydispersity of the system, %

Magnetite particles can develop charges due to the protonation and deprotonation reactions of Fe≡OH surface sites, such as ≡Fe-OH + H^+ <=> ≡Fe-OH_2^+ and ≡Fe-OH + OH^- <=> ≡Fe-O- + H_2O, respectively. The net proton surface excess (Δnσ), which is related to the surface charge density, was determined. Potentiometric acid–base titration was performed in the pH range 3–11 at 0.005, 0.05, and 0.5 M KCl concentrations. The Δnσ for bare magnetite in the function of pH can be seen in Fig. 2.

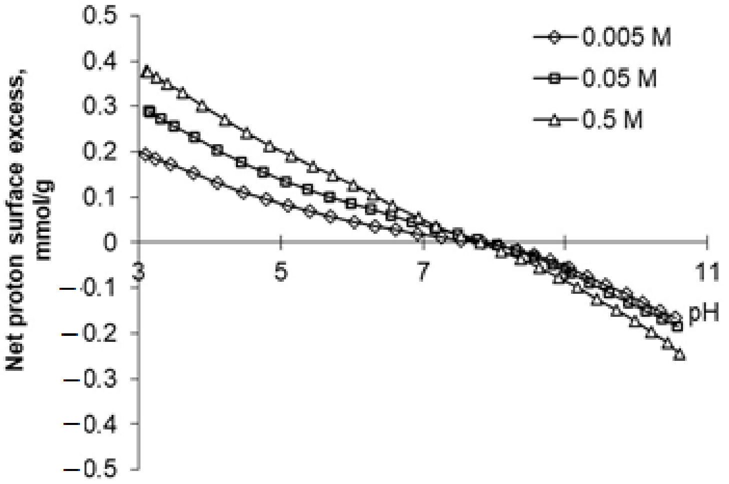

Figure S2. pH-dependent surface charging of magnetite at different KCl concentrations. The experimental points were calculated from the material balance of H+/OH− during equilibrium acid–base titration

H+-ions accumulate on the surface at pHs lower than the point of zero charge (PZC), therefore the surface charge is positive, while magnetite particles are negatively charged in alkaline solutions above the pH of the PZC. The experimental PZC value of magnetite is pH~7.8. The surface charge density increases with increasing KCl concentrtion due to the charge screening effect of the salt.

Below pH~3 and above pH ~10.5, the dissolution of metal oxides may occur. The solubility of magnetite, owing to its Fe(II) content, is usually higher than that of pure Fe(III) oxides. Nevertheless, the concentration of dissolved Fe(III) species is small (i.e. not greater than ~10–5 mol/dm3 within pH range from ~4 to ~10 [37]) and thus the dissolution of particles can be ignored within titration pH range.

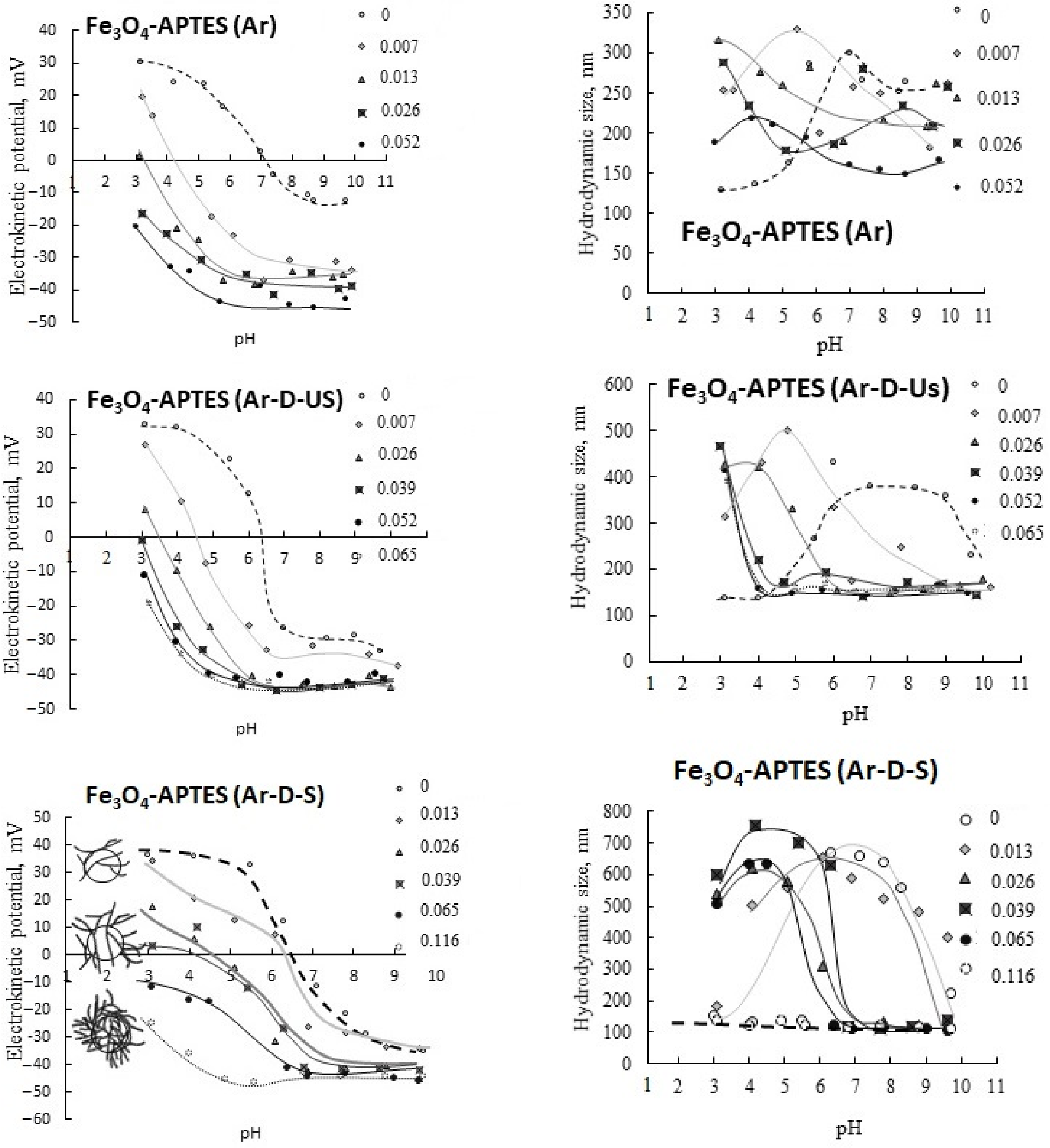

Figure S3. a) Effect of humic preparation (g/g) adsorption on the pH-dependent zeta potential of Fe_3O_4±StdDev; b) Average hydrodynamic size of NPs±.StdDev.
